# Describing and understanding behavioral responses to multiple stressors and multiple stimuli

**DOI:** 10.1002/ece3.2609

**Published:** 2016-11-29

**Authors:** Robin Hale, Jeremy J. Piggott, Stephen E. Swearer

**Affiliations:** ^1^ School of BioSciencesUniversity of MelbourneParkvilleVICAustralia; ^2^ Department of ZoologyUniversity of OtagoDunedinNew Zealand; ^3^ Center for Ecological ResearchKyoto UniversityOtsuJapan

**Keywords:** antagonism, cue, ecological surprise, effect size, interaction, multiple stressor, sensory pollution, synergism

## Abstract

Understanding the effects of environmental change on natural ecosystems is a major challenge, particularly when multiple stressors interact to produce unexpected “ecological surprises” in the form of complex, nonadditive effects that can amplify or reduce their individual effects. Animals often respond behaviorally to environmental change, and multiple stressors can have both population‐level and community‐level effects. However, the individual, not combined, effects of stressors on animal behavior are commonly studied. There is a need to understand how animals respond to the more complex combinations of stressors that occur in nature, which requires a systematic and rigorous approach to quantify the various potential behavioral responses to the independent and interactive effects of stressors. We illustrate a robust, systematic approach for understanding behavioral responses to multiple stressors based on integrating schemes used to quantitatively classify interactions in multiple‐stressor research and to qualitatively view interactions between multiple stimuli in behavioral experiments. We introduce and unify the two frameworks, highlighting their conceptual and methodological similarities, and use four case studies to demonstrate how this unification could improve our interpretation of interactions in behavioral experiments and guide efforts to manage the effects of multiple stressors. Our unified approach: (1) provides behavioral ecologists with a more rigorous and systematic way to quantify how animals respond to interactions between multiple stimuli, an important theoretical advance, (2) helps us better understand how animals behave when they encounter multiple, potentially interacting stressors, and (3) contributes more generally to the understanding of “ecological surprises” in multiple stressors research.

## 1 Introduction

The need to better understand the effects of multiple stressors is cited as one of the most important questions in conservation and applied ecology (e.g., Zeidberg & Robison, 2007). Of particular interest and concern are situations where multiple stressors interact to create “ecological surprises” in the form of complex, nonadditive effects such as synergisms (amplified combined effects) or antagonisms (reduced effects) (Folt, Chen, Moore, & Burnaford, 1999). Complex interactions between stressors are likely to be highly prevalent (e.g., Crain, Kroeker, & Halpern, 2008; Jackson, Loewen, Vinebrooke, & Chimimba, 2016), yet the mechanisms for such responses remain largely unexplored.

Altering their behavior is one of the main ways that animals respond to environmental change (Wong & Candolin, 2015), and can help them cope, even thrive, under new environmental conditions (Sih, 2013). However, stressors (“any natural or anthropogenic pressure that causes a quantifiable change, whether positive or negative, in biological response”—Côté, Darling, & Brown, 2016) acting in concert can compromise the behavior of animals and ultimately result in changes in community interactions (Francis, Ortega, & Cruz, 2009). While the effects of interacting stressors have been studied in some contexts (e.g., the effects of multiple predators—Sih, Englund, & Wooster, 1998), often only the individual effects of stressors on animal behavior are studied, and usually effects on one sensory modality only. However, stressors commonly covary and may interact in complex ways, requiring researchers to examine how animals respond to the full factorial combinations of sensory stressors they experience in nature (Halfwerk & Slabbekoorn, 2015). This will necessitate a rigorous and systematic approach to quantify how animals behave when exposed to the independent and interactive effects of multiple stressors.

We illustrate a robust, systematic approach for understanding behavioral responses to multiple stressors based on integrating two pre‐existing frameworks: schemes to classify interactions in multiple‐stressor research (Crain et al., 2008; Piggott, Townsend, & Matthaei, 2015b), and qualitative methods for viewing interactions between multiple stimuli in behavioral experiments (Munoz & Blumstein, 2012; Partan, 2004; Partan & Marler, 1999). We firstly introduce the two frameworks, highlighting their conceptual and methodological similarities, before demonstrating how they can be unified. We then use four case studies to illustrate how this unification could change our interpretation of behavioral responses to interactions between stimuli or stressors and to guide efforts in ameliorating the effects of multiple stressors. We highlight the benefits of this approach in terms of: (1) providing a systematic method to examine interactions in behavioral ecology, an important theoretical advance, (2) improving our understanding of behavioral responses to multiple stressors, and (3) contributing to an improved understanding of interactions in multiple‐stressor research more generally.

## 2 Characterizing multiple‐stressor interactions

Interpreting how animals respond to multiple stressors depends on firstly understanding their responses to each in isolation. Four responses to two hypothetical stressors (A and B) are possible: (1) both affect animals in the same direction (e.g., A and B both cause mortality), (2) their effects are opposing (e.g., A reduces but B increases growth), (3) one stressor elucidates a response but the other does not (e.g., A causes mortality, B has no effect) and (4) neither results in a significant response. Complex interactions between stressors are possible, and understanding these is crucial for elucidating stressor mechanisms and separating effects based on severity (Piggott et al., 2015b). The next step therefore is to consider how responses to individual stressors change when they co‐occur. Broadly speaking, if stressor A reduces response by “a” and stressor B by “b,” the cumulative effect of A + B can be additive (=a + b), antagonistic (<a + b) or synergistic (>a + b). Crain, Kroeker, & Halpern (2008) provide a wealth of examples of different interaction types in marine ecosystems such as the synergies between UV radiation and temperature or toxins, where negative effects are considerably stronger in concert, or antagonism between salinity and temperature/toxins where the interactive effects are weakened.

**Figure 1:**
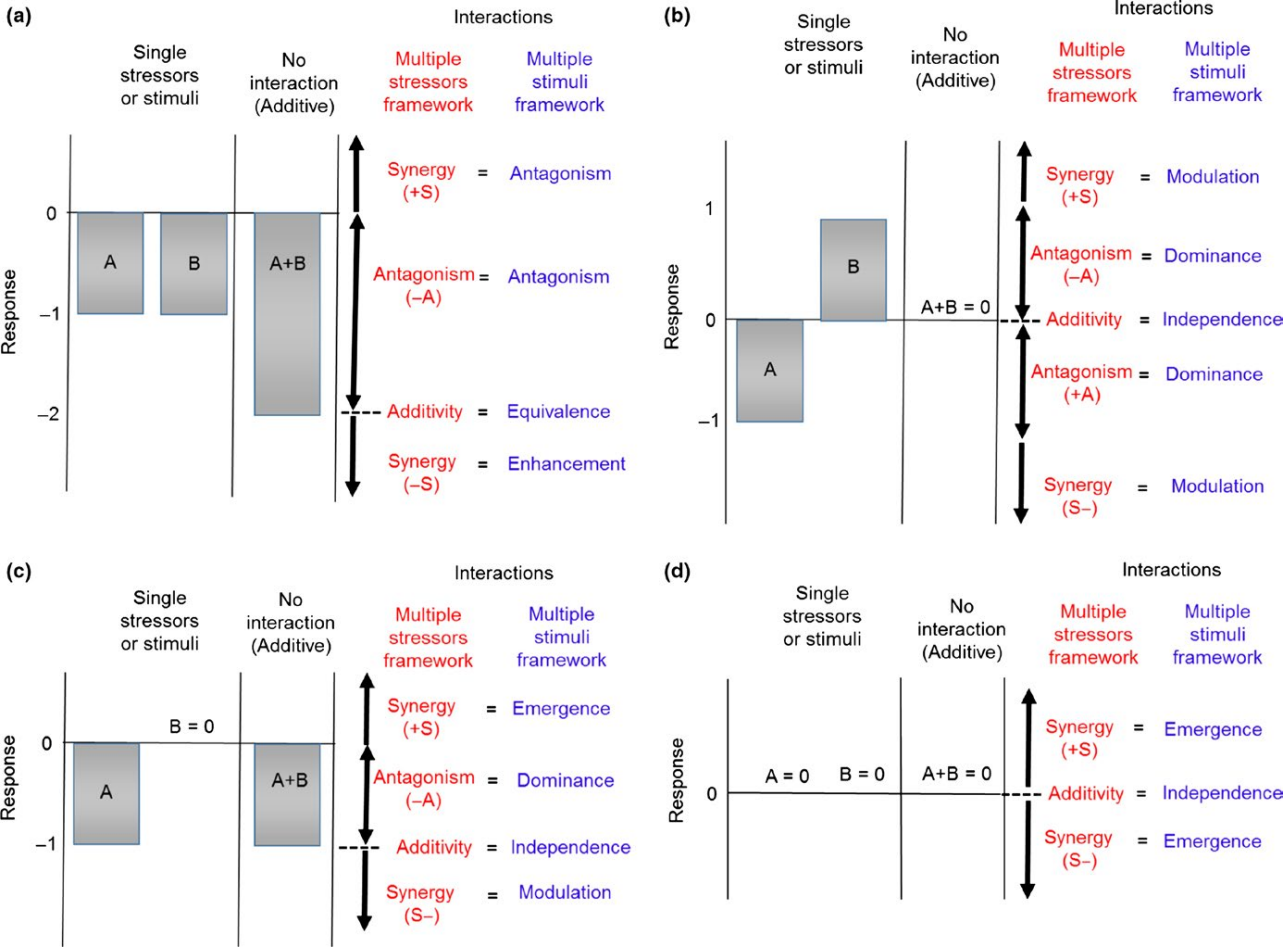
A conceptual framework for classifying behavioral response to multiple stressors based on the direction and magnitude of their individual and interactive effects. The plots show the four main ways that animals can respond to two stressors, when: (a) both have significant effects in the same direction, (b) both have significant effects in different directions, (c) one causes a significant effect but the other no response, and (d) when both have no significant response. The gray bars show the response to each stressor, and the expected value of the response when there is no interaction (i.e., effects are additive). The red text illustrates how interactions would be classified following Piggott et al. (2015b) based on calculations of effect sizes for individual and interactive effects and their associated 95% confidence intervals. Blue text illustrates how interactions would be classified following Munoz & Blumstein (2012). The integration of these two schemes is based on the six conditions in Table 1. Table S1 outlines classifications for all interaction types (e.g., when the individual effects of A and B shown in (a) are both positive rather than negative)

Crain et al. (2008) outlined a conceptual framework for interpreting interaction types between stressors based on characterizing the strength and direction of effect sizes associated with individual and interactive effects. They conceptualized interactions based on the direction of three broad categories of individual effects: double‐positive, double‐negative, and opposing. While identifying synergism or antagonism is generally straightforward when stressors operate in the same direction, it can be more difficult when individual effects are opposing (i.e., A and B lead to positive and negative responses, respectively) or combined effects are reversed (i.e., A and B both lead to positive responses individually but a negative responses in combination). To interpret these more complex interactions, Piggott et al. (2015b) proposed a revised classification, where the following interactions are possible (Figure 1; Table S1):

1. *Additive* (e.g., no significant interaction in an ANOVA model), whereby the interaction represents the sum of individual effects;
2. *Antagonistic* interactions that are not additive (e.g., a significant ANOVA interaction term) and weaker than expected (<a + b and < |a| or |b|). These can be either *positively* (+A; less positive than predicted additively) or *negatively* (‐A; less negative than predicted additively) *antagonistic* depending on the direction of individual effects.
3. *Synergistic* interactions that are not additive (e.g., a significant ANOVA interaction term) but are stronger than expected (>a + b and > |a| or |b|). These can be either *positively* (+S more positive than predicted) or *negatively* (−S more negative than predicted) s*ynergistic*, depending on the direction of individual effects.

## 3 Characterizing how animals respond behaviorally to multiple stimuli

Animals use a diversity of visual, olfactory, and auditory information (e.g., environmental cues) to make critical life history decisions (e.g., communication, resource selection), and there has been significant research describing behavioral responses to these stimuli using one or more sensory modalities (Hebets & Papaj, 2005; Ward & Mehner, 2010). Responding to multiple stimuli is likely common among animals as it reduces the risk of missing opportunities to maximize fitness (Munoz & Blumstein, 2012). Although the behavioral responses of animals to multiple stimuli can be characterized using the same reasoning as that outlined above for multiple stressors, no systematic methods exist to do so.

How animals respond to multiple stimuli will depend on how individual components interact, and the efficacy of information transfer through the environment (Hebets & Papaj, 2005). Analogous to interactions among multiple stressors, animals may respond differently depending on whether they encounter stimuli alone or in combination (Hebets & Papaj, 2005; Munoz & Blumstein, 2012; Partan, 2004; Partan & Marler, 1999, 2005). Partan and Marler (1999, 2005) proposed a conceptual framework that provides an intuitive basis for visualizing and characterizing animal responses to multiple signals. This framework has also been adapted to classify multisensory stimuli based on behavioral responses to individual components in isolation and in combination (Munoz & Blumstein, 2012) (hereafter the “multiple stimuli framework”).

Using this framework, stimuli are initially classified in terms of whether they invoke similar individual responses in the same direction (“redundant”) or responses in different directions (“nonredundant”), before these groups are further classified based on how individual responses interact.

Redundant interactions can be further classified in three ways (Munoz & Blumstein, 2012). First, there may be no interaction; that is, responses are comparable to when stimuli are encountered in isolation (“equivalence”). For example, western mosquitofish (*Gambusia affinis*) use visual and chemical cues additively to avoid predators (Smith & Belk, 2001). Second, responses may be more intense (“enhancement”). For example, star gobies (*Asterropteryx semipunctatus)* respond more strongly when offered visual and chemical stimuli than when offered these alone (McCormick & Manassa, 2008). Third, responses may be weaker (“antagonism”). For example, sagebrush lizards (*Sceloporus graciosus)* respond more weakly when exposed to visual and chemical stimuli simultaneously than when exposed to either stimulus alone (Thompson, Bissell, & Martins, 2008).

Four outcomes are possible from interactions between nonredundant components (Munoz & Blumstein, 2012; Partan & Marler, 1999, 2005). First, the response includes only the individual responses to each component (“independence”), as demonstrated by the responses of tropical wandering spiders (*Cupiennius salei*) to chemical and vibratory signals during courtship (Rovner & Barth, 1981). Second, the response may only contain the individual responses to one of the components (“dominance”). For example, visual cues dominate chemical cues in the predator avoidance behavior of mosquitofish (*Gambusia holbooki*) (Ward & Mehner, 2010). Third, one component influences the response to another component, so the response to the combination of stimuli is of the same type but is changed in some degree (“modulation”). Fourth, the combined response may be qualitatively different from the response to each stimuli individually (“emergence”). For example, tungara frogs *(Engystomops pustulosus*) are attracted to a signal containing both acoustic and visual stimuli, neither of which elicit a response in isolation (Taylor & Ryan, 2013).

## 4 Understanding and classifying interactions between environmental stimuli

The multiple stimuli frameworks provide an intuitive way to visualize how animals respond to two potentially interacting stimuli but not to quantitatively classify interactions. A major issue is that behavioral researchers commonly use null hypothesis testing approaches that do not provide information about the size of any effects of interest or precision in their estimates (Nakagawa & Cuthill, 2007). Yet classifying interactions in the multiple stimuli framework depends on such information, as two simple contrasts demonstrate. First, assume that an animal responds positively to two stimuli independently, and these interact (i.e., all main effects and the interaction are statistically significant in a two‐factor ANOVA). Plotting the data will enable us to determine whether this interaction is weaker (i.e., “antagonism”) or stronger (i.e., “synergism”) than the independent effects. Now assume that an animal exhibits positive and negative responses to two stimuli independently. If these two stimuli interact, it is impossible to distinguish between cases of dominance or modulation without quantitatively measuring the magnitude of the independent and interactive effects to these stimuli.

## 5 Unifying the multiple stressors and multiple stimuli frameworks

The aims of the “multiple stimuli” and “multiple stressors” frameworks are conceptually the same—to examine potential responses to multiple stressors (or stimuli) in isolation, and to then assess whether and how these responses change when they are offered simultaneously. The same methods are also commonly used in both types of studies, a two‐factor factorial experiment with four treatments: a control, stressor/stimuli A, stressor/stimuli B, and the interaction (i.e., stressor/stimuli A × stressor/stimuli B). While Partan and Marler (2005) avoided using “additive” and “synergistic” in their original framework due to ambiguities with how these terms have been used previously, both types of studies are commonly analyzed using analysis of variance (ANOVA) models, where the null hypothesis is that an interaction will be additive (Piggott et al., 2015b), and a statistically significant interaction term provides evidence of nonadditivity.

Integrating the two frameworks would couple the rigorous classification scheme developed for multiple stressors with the advantages of the multiple stimuli framework as an intuitive way to visualize interactions. It is important to note that we are not suggesting new terms here, just that the terminology and methodology outlined for the multiple stressors framework can be applied to classify interactions between stimuli. Based on six conditions (Table 1), we demonstrate what the respective classifications would be under the two schemes (Figure 1).

**Table 1:** Reasoning underpinning the conceptual framework for classifying behavioral responses to multiple stressors based on the direction and magnitude of their individual and interactive effects (Figure 1). Six conditions underpin the integration of schemes for classifying interactions between multiple stressors (Piggott et al., 2015b; hereafter P) and multiple stimuli (Munoz & Blumstein, 2012, MB)

	Condition details
1	Double‐negative/ double‐positive interactions (P) are analogous to redundant stimuli (MB). All other interactions are analogous to nonredundant stimuli.
2	Additive interactions (independence—P) are analogous to equivalence and independence for redundant and nonredundant stimuli, respectively (MB).
3	Double‐negative interactions (Figure 1a) that are more negative than additive are analogous to enhancement (MB), and less negative than additive to antagonism (MB). The latter can be further classified as antagonistic or synergistic (i.e., reversal or “mitigating synergism”) (P) based on the strength of the interaction. The same reasoning applies to double‐positive interactions except the direction of individual effects is in the opposite direction (i.e., more positive than additive = enhancement, less positive = antagonism);
4	For opposing interactions (Figure 1b), antagonism (P) is analogous to dominance (MB), and synergism (P) to modulation (MB). Dominance occurs when the confidence intervals for the interaction and individual effect in the same direction overlap, and modulation when the interaction confidence interval is greater.
5	For negative or positive (i.e., non‐neutral) versus neutral interactions (Figure 1c), all antagonistic interactions are classified as dominance (MB), all synergistic interactions in the same direction as the non‐neutral stimuli as modulation, and in the opposite direction as emergence.
6	Emergence (MB) occurs when any interaction between two neutral individual effects is not additive (Figure 1d).

## 6 Methodology to assess and classify interactions

The analytical methods to classify interactions following Piggott et al. (2015b) involve calculating the individual, main, and interactive effect sizes (Hedges *d*) and their associated confidence intervals following methods used for factorial meta‐analysis (Gurevitch, Morrison, & Hedges, 2000). Individual effects compare the effects of each treatment separately to the control, that is, in the absence of the other treatment. Main effects are analogous to the main effect in a two‐way ANOVA and compare the mean performance in the two treatments when the agent is present versus the two treatments where the agent is absent.

By calculating 95% confidence intervals for the interaction effect size, interactions are initially classified as additive (95% CI overlaps zero), or not (95% CI does not overlap zero). All nonadditive interactions can then be classified based on the direction and magnitude of the individual and interactive effects (following Piggott et al., 2015b, Fig. 1, Table S1). This approach provides information about both the magnitude of an effect of interest and precision in its estimate and allows precise differentiation of interactions. For example, it is possible to differentiate between negative synergistic and positive antagonistic effects (e.g., Piggott et al., 2015b, Fig. 2 iii) when both individual effects are positive, but the interaction is negative.

**Figure 2:**
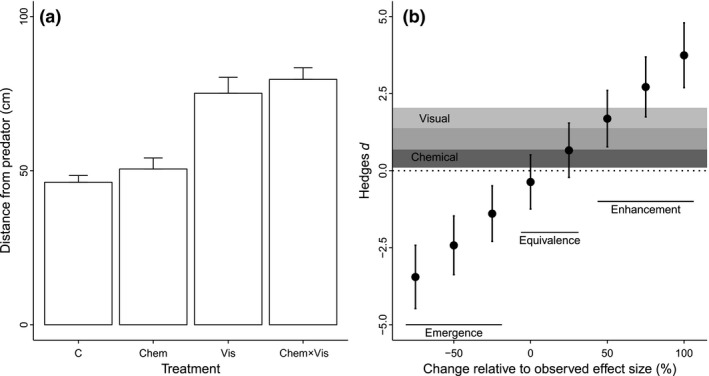
Antipredator behavior in western mosquitofish on the basis of visual (Vis) and chemical (Chem) cues. (a) Raw data extracted from Smith and Belk (2001, fig. 1 small fish only). The control (C) represents the response of mosquitofish to a satiated predator fed on a chironomid diet, and the interaction (Chem × Vis) the response to hungry predators fed on a mosquitofish diet. (b) Effect sizes (Hedges *d*) for individual and interactive effect sizes calculated as described in the text, and interactions classified following Figure 1 and Table 1. The shaded regions are the 95% confidence intervals for the individual effects of visual (light gray), and chemical (dark gray) stimuli; medium gray indicates overlap between the confidence intervals. The black error bars indicate 95% confidence intervals for the interaction, based on different effect sizes, with 0 on the abscissa corresponding to the effect size observed in the study, and values less than and greater than 0 representing simulated effect sizes smaller or larger than those observed, respectively. The black text and horizontal lines demonstrate how classifications of the interaction would change relative to changes in effect size

## 7 Case studies integrating the multiple stressors and stimuli frameworks

We use three case studies to illustrate how using the approach proposed above could help us interpret how animals respond to multiple, interacting stimuli. We extracted the raw data from each of these studies using Web Plot Digitiser (http://arohatgi.info/WebPlotDigitizer/app) and calculated effect sizes and their associated confidence intervals for the individual and interactive effects (annotated code for these calculations is included as Supporting Information). For each study, we then selected a range of smaller and larger effect sizes for the interaction between stimuli than was observed, and recalculated all effect sizes and confidence intervals. We did this to illustrate potential changes in how interactions are classified if stronger or weaker effects had been observed. A full sensitivity analysis examining the effects of all parameters from each study was beyond the scope of the study (i.e., number of samples, mean, and standard deviation for each of the four treatments: control, stimulus A alone, stimulus B alone, stimulus A × stimulus B).

### 7.1 Antipredator behavior in western mosquitofish

Our first example is the study of antipredator behavior in western mosquitofish (*Gambusia affinis*) (Smith & Belk, 2001). Groups of mosquitofish were exposed to chemical (predator diet) and visual (behavioral differences between hungry and satiated predators) cues from the predatory green sunfish (*Lepomis cyanellus)*. Mosquitofish maintained a greater mean distance from predators fed on mosquitofish than those fed on chironomids (a positive response to chemical stimuli), and also from hungry rather than satiated predators (a positive response to visual stimuli), but there was no interaction between chemical and visual stimuli (Figure 2a).

Our analysis (Figure 2b) is consistent with that presented in Smith and Belk (2001); that is, the effects of visual and chemical cues are additive. Our results illustrate that: (1) both cue types had confidence intervals greater than zero (i.e., a “double‐positive” interaction—Table S1) and were thus redundant, and (2) the interaction confidence interval spans zero (i.e., is not different from additive). The interaction in this case therefore represents an example of equivalence (Table S1). An interaction effect size smaller (<75% than the observed) or larger (>50% the observed) would lead to classifications of emergence and enhancement, respectively.

### 7.2 Prey assessment by wolf spiders

**Figure 3:**
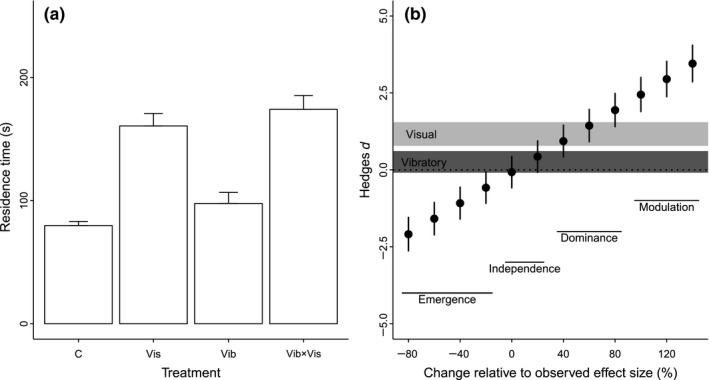
Multimodal signaling in the wolf spider. (a) Raw data extracted from Persons and Uetz (1996, fig. 2). C: control, Vis: visual stimuli, Vib: vibratory stimuli, Vib × Vis: both vibratory and visual stimuli (b) Summary of classification of interactions based on effect size and confidence intervals for nonredundant cues. Description of figure follows Figure 2, although note no overlapping confidence intervals for the individual effects of visual and vibratory stimuli

Our second example is the study by Persons and Uetz (1996) examining the responses of wolf spiders (*Schizocosa ocreata*) to visual and vibratory stimuli. The authors measured the residence time (time spent watching prey) of spiders when exposed to four treatments: a control, a visual stimulus (a live cricket), a vibratory stimulus (substrate‐borne oscillations from crickets), and both visual and vibratory stimuli in combination. Wolf spiders spent longer in patches when exposed to visual stimuli and the combination of visual and vibratory stimuli than either vibratory stimuli alone or the control (Figure 3a).

Partan and Marler (2005) classified the study by Persons and Uetz (1996) as an example of modulation. Our analysis (Figure 3b) illustrates that visual stimuli elucidated a positive response (i.e., “visual” confidence interval > 0), but spiders did not respond to vibratory stimuli (i.e., “vibratory” confidence interval spans 0). Therefore, the two stimuli are nonredundant and represent a “positive neutral” interaction type (Table S1). The interaction confidence spanned zero (i.e., was not significantly different from additive), which means that this represents a case of independence between visual and vibratory cues (Table S1). For “positive neutral” interactions, dominance occurs when the strength of the interaction and the stronger individual effect are similar, and modulation when the interaction is significantly stronger than the strongest individual effect. Our results demonstrate that dominance would be observed if the effect size was 40%–80% greater than observed, and modulation if the effect size was increased by >80%.

### 7.3 Domestic gulls avoiding insect warning displays

**Figure 4:**
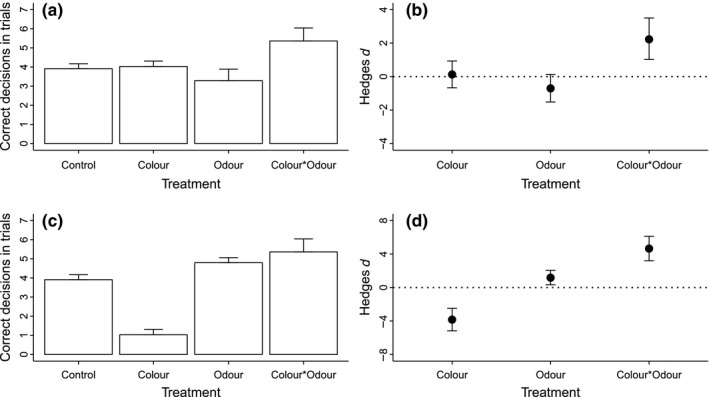
The responses of domestic chicks (*Gallus gallus domesticus*) to visual (yellow color) and chemical (odors from pyrazine) insect warning displays when exposed to food items. (a) Raw data extracted from Rowe and Guilford (1996, fig. 2). We used their green visual treatment with no odor as the “control”, their yellow treatment with no odor as the “color” treatment, their green with pyrazine odor as the “odor” treatment, and their yellow with odor as “color × odor”. The response variable here is the number of correct decisions per trial, in terms of avoiding unpalatable food items. For reference, four correct decisions indicates no discrimination, 8 represents complete discrimination, and 0 represents incorrect discrimination. (c) Hypothetical example illustrating how the classifications of interactions between odor and color would change if one elicits a negative response in isolation (odor) and the other a positive response (color). In panels b and d, we present effect sizes (Hedges *d*, calculated as per Figures 2, 3) for the individual and interactive effects of Color and Odor

Our third example is the study examining how birds (domestic chicks *Gallus gallus domesticus*) respond to warning signals of toxic insects consisting of pyrazine odors and conspicuous yellow coloring (Rowe & Guilford, 1996). One group of chicks experienced four green palatable and four yellow unpalatable (soaked in quinine and mustard) prey, while another experienced the opposite, that is, yellow unpalatable, green palatable. Half of the birds in each group were tested in the presence of pyrazine and the others without. The numbers of correct responses (avoidance of unpalatable food) to the following treatments were recorded: (1) green unpalatable items with no pyrazine (“control” in Figure 4), (2) yellow unpalatable items with no pyrazine (representing the individual effect of yellow color: “color” in Figure 4), (3) green unpalatable food with pyrazine (the individual effect of pyrazine: “odor” in Figure 4), and (4) yellow unpalatable food with pyrazine (“color × odor”).

Chicks did not respond to the individual effects of either color or odor (Figure 4a), but correctly avoided yellow unpalatable food items when these also contained pyrazine odors (“color × odor treatment”). This represents a case of emergence, when animals exhibit a significance response to a combined stimuli but not to its individual components (Partan & Marler, 2005). Our analysis concurs with this conclusion, the effect size for the “color × odor” treatment being significantly greater than zero, while neither of the individual effects are (Figure 4b).

We also use this example to demonstrate how the classification of this interaction would change if the individual responses to color and odor were observed to be acting in opposing directions, for example, if chicks made fewer correct decisions in response to yellow food items but more correct responses when pyrazine was present (Figure 4c). Our analysis of this hypothetical example (Figure 4d) demonstrates a case of modulation—the effects of color and odor are negative and positive, respectively, and the combined color × odor effect is larger than the effects of odor alone (Figure 1b). Dominance would occur if the confidence intervals for the color × odor and odor treatments overlap.

## 8 What do these examples tell us?

Our first example illustrates that it is straightforward to classify interactions using the results of ANOVA or equivalent models, and appropriate summaries of the data, when individual effects are in the same direction. However, it is considerably more difficult when some of the individual or interactive effects are marginally significant (e.g., Example 2). When individual effects are in opposite directions, it is impossible to classify interactions (e.g., distinguishing modulation from dominance in Example 3) without calculating confidence intervals for the individual and interactive effects as per the multiple stressors approach.

## 9 Unifying the multiple stressors and multiple stimuli frameworks can guide the management of multiple stressors

Our approach can help guide the management of multiple stressors as demonstrated in our fourth case study. Chan, Giraldo‐Perez, Smith, and Blumstein (2010) assessed the effects of anthropogenic noise on predator risk assessment by Caribbean hermit crabs (*Coenobita clypetus*), showing that crabs allowed a simulated predator to approach closer (measured as hiding initiation distance—HID) in the presence of playbacks of motorboat noise relative to silent controls. In a further experiment, simulated predators were able to approach crabs more closely when flashing lights were added as a second stressor, providing evidence that crabs might be reallocating some of their finite attention, distracting them from responding to an approaching threat.

**Figure 5:**
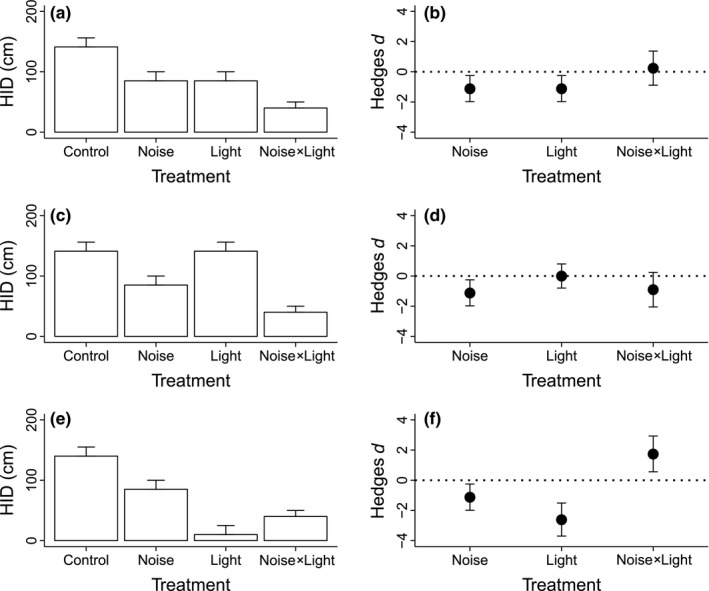
The effects of anthropogenic noise on predator risk assessment in Caribbean hermit crabs (*Coenobita clypeatus*), with the response measured as hiding initiation distance (HID—cm), the distance that a predator can approach before crabs hide in their shells. Raw data extracted from Chan et al. (2010, fig. 2e) for the “noise” and “noise × light” treatments in panel a, which represent exposure to boat noise alone, and boat noise simultaneously with flashing lights. We used their Figure 2b, and the reduction in HID between their control (“silence”) and “noise” treatment as an indication of the likely control value. In the original study, HID in response to flashing lights alone was not quantified. We explore how the noise × light interaction would be classified if: (a) noise and lights lead to comparable reductions in HID (an “additive” interaction—Figure 1, Table S1), (b) noise reduces HID in isolation but lights do not (also an additive interaction), and (c) both reduce HID, but light has significantly stronger effects than noise (an antagonistic interaction). In panels b,d,f, we present effect sizes (Hedges *d*, calculated as per Figures 2, 3, 4) for the individual and interactive effects of noise and light for each of these three scenarios

While Chan et al. (2010) quantified how crabs respond to boat noise alone and in combination with flashing lights, they did not examine responses to flashing lights in isolation. We extracted data from the original study as above, and our subsequent analyses (Figure 5) highlight that different individual responses to light could modify the noise × light interaction, in turn affecting whether reducing light or noise exposure is likely to be the most appropriate management option. For example, noise and light might both reduce the distance a predator can approach before a crab commences antipredator behavior with comparable effect sizes (Figure 5a), leading to an additive interaction (Figure 5b). If so, then reducing either the effects of light or noise in isolation is likely to be ineffective. However, if crabs are not affected by lights in isolation (Figure 5c,d), reducing noise exposure should be the priority. Alternatively, light exposure alone could reduce HID more so than either noise alone or the interactive effects of both stressors (Figure 5e). If so, an interaction that is more positive than additive represents an antagonistic relationship between lights and noise (Figure 5f) and would indicate that crabs are likely to respond more strongly to actions that ameliorate the effects of light. Although this last scenario might be unlikely in the specific case of the Chan et al. (2010) study, what these three scenarios illustrate is how identifying the size and magnitude of the individual and interactive effect sizes associated with multiple stressors could help managers determine whether one or multiple stressors need to be ameliorated, and which should be the priority if the former is true.

## 10 Conclusions

Our study represents an important contribution toward disentangling the mechanistic pathways by which multiple stressors interact in ecosystems. Because community‐level responses to multiple stressors are likely manifested through a complex interplay of physiological, ecological, and behavioral interactions, determining the magnitude and direction of behaviorally mediated responses can help elucidate stressor responses at higher organizational levels. For example, Piggott, Townsend, and Matthaei (2015a) demonstrated how stream invertebrate drift behavior drove powerful community shifts in response to fine sediment, but less so for temperature or nutrient stressors. Identifying cases of behavioral “emergence” is of particular interest in explaining “ecological surprises,” that is, unexpected responses such as “reversals” (Jackson et al., 2016) or “mitigating synergisms” (Piggott et al., 2015b) that are increasingly appearing in the multiple stressors literature.

Many ecosystems are stressed in multiple, potentially interacting ways. For example, the “urban stream syndrome” describes the raft of physical, chemical, and biological changes that occur in aquatic ecosystems in response to urbanization (Walsh et al., 2005). For many animals, behavioral changes are often the first response to human‐altered conditions (Wong & Candolin, 2015). While some pollutants might affect behavior in an additive fashion, other more complex interactions are likely—this possibility is poorly understood, especially when stressor effects are multisensory (Halfwerk & Slabbekoorn, 2015). Work is needed moving beyond examining the effects of one stressor to assessing how animals respond to a range of stressors in the presence of ecologically important signals and cues (Halfwerk & Slabbekoorn, 2015). Our framework is a useful tool that can be used in this process, to help quantitatively assess the nature of responses to individual and interactive stressors. Identifying interactions can help prioritize conservation actions by identifying which stressor(s) should be the priority for producing the most beneficial outcomes for animals, as our final case study illustrates. While identifying the effects of cumulative stressor impacts will undoubtedly be complicated, the reasoning used here (i.e., quantifying individual responses to stressors and how these change in combination) can be extended from the relatively straightforward situation where two stressors are present to help guide efforts examining how animals respond to three or more stressors.

Adopting a more systematic approach, especially the calculation and comparison of effect sizes, will improve attempts to predict behavioral responses to environmental changes and their consequences for animals (Blumstein, 2015). How animals select habitats is likely to be a key determinant of whether they respond adaptively or not to habitat change (Hale, Treml, & Swearer, 2015), and the likelihood of maladaptive behavior will depend on the nature of the interaction between sensory stimuli and which signals and cues are impacted. Species are likely to be more susceptible to making such errors when dominant stimuli become misleading, than if they use independent and equivalent stimuli.

Behavioral ecologists are becoming increasingly interested in studying how animals respond to combinations of environmental stimuli, potentially using multiple sensory modalities. The multiple stimuli frameworks provide important conceptual tools for visualizing the potential ways that different stimuli interact. Our approach provides a rigorous and systematic means to quantitatively classify and interpret these interactions and can be a useful tool to help improve our understanding of how animals use environmental stimuli to reduce uncertainty when making key life history decisions, and respond to multiple stressors. Underpinning this effort is the need for researchers to move beyond simple hypothesis testing methods and *p*‐values, to be complemented by making effect size and confidence interval data available for transparency and meta‐analysis. This suggestion has been made previously (e.g., Nakagawa & Cuthill, 2007), but once a sufficiently large pool of studies are undertaken using our approach, it will be possible to investigate the potential commonality of different interaction types, for example, via formal meta‐analyses (e.g., Jackson et al., 2016). Some interaction types are likely to be more common in different contexts (e.g., nonredundant cues in mate choice—Candolin, 2003), but such comparisons would shed light on whether animals use multimodal and multicomponent stimuli in similar ways (Partan, 2013), and help us better understand the effects of anthropogenic disturbances on animal behavior.

## Conflict of Interest

We have no competing interests.
