# Supplementary material for: Describing and understanding behavioral responses to multiple stressors and multiple stimuli
Source: Ecol Evol. 2016 Nov 29;7(1):38–47. doi: 10.1002/ece3.2609 (PMC5214703; doi:10.1002/ece3.2609)
Supplement: Supplementary file 1 [file ECE3-7-38-s001.docx]

Supplementary Table S1 Outline of conceptual framework for classifying interactions based on the direction and magnitude of their individual and interaction effects (columns a – f), adapted from Piggott, Townsend & Matthaei (2015b). Linking schemes for classifying the type of interactions for multiple stressors following Piggott, Townsend & Matthaei (2015b) (columns e-f) and multiple stimuli following Munoz and Blumstein (2012) (columns g-h). The direction of individual stimuli effects (a) or (b) and interaction effects (a+b) are coded as negative (-), positive (+) or neutral (0). Double symbols (+ +, --) indicate the direction of a cumulative effect (a + b) that is greater than the sum of individual effects and greater than any individual effect in the same direction, or has an interaction effect that is greater than both in absolute terms. Directional interaction classes (Multiple stressors classification Interaction type) are additive (AD), +synergistic (+S), -synergistic (-S), +antagonistic (+A) and –antagonistic (-A)

|  | | | | Multiple stressors classification | | Multiple stimuli classification | |
| --- | --- | --- | --- | --- | --- | --- | --- |
| (a) Interaction type | (b) Individual effects of a | (c) Individual effects of b | (d) Interactive effects of a+b | (e) Interaction classification | (f) Example (a+b=Interaction classification) | (g) Redundant (R)/non-redundant (NR) | (h)  Interaction classification |
| Double positive | + | + | ++ | +S | 1+1=2<(+S) | R | Enhancement |
|  | + | + | + | +S | 1+1=2<(+S) | R | Enhancement |
|  | + | + | 0 | AD | 1+1=2(AD) | R | Equivalence |
|  | + | + | - | +A | 1+1=0≤(A+)<2 | R | Antagonism |
|  | + | + | -- | -S | 1+1=(S-)<0 | R | Antagonism |
|  |  |  |  |  |  |  |  |
| Double negative | - | - | ++ | +S | -1+-1=0<(S+) | R | Antagonism |
|  | - | - | + | -A | -1+-1=-2<(A-)≤0 | R | Antagonism |
|  | - | - | 0 | AD | -1+-1=-2(AD) | R | Equivalence |
|  | - | - | - | -S | -1+-1=(S-)<-2 | R | Enhancement |
|  | - | - | -- | -S | -1+-1=(S-)<-2 | R | Enhancement |
|  |  |  |  |  |  |  |  |
| Opposing | + | - | ++ | +S | 1+-1=1<(+S) | NR | Modulation |
|  | + | - | + | -A | 1+-1=0<(A-)≤1 | NR | Dominance |
|  | + | - | 0 | AD | 1+-1=0(AD) | NR | Independence |
|  | + | - | - | +A | 1+-1=-1≤ (A+)<0 | NR | Dominance |
|  | + | - | -- | -S | 1+-1=(S-)<-1 | NR | Modulation |
|  |  |  |  |  |  |  |  |
| Opposing | - | + | ++ | +S | -1+1=1<(+S) | NR | Modulation |
|  | - | + | + | -A | -1+1=0<(A-)≤1 | NR | Dominance |
|  | - | + | 0 | AD | -1+1=0(AD) | NR | Independence |
|  | - | + | - | +A | -1+1=-1≤ (A+)<0 | NR | Dominance |
|  | - | + | -- | -S | -1+1=(S-)<-1 | NR | Modulation |
|  |  |  |  |  |  |  |  |
| Negative neutral | - | 0 | ++ | +S | -1+0=0<(+S) | NR | Emergence |
|  | - | 0 | + | -A | -1+0=-1<(A-)≤0 | NR | Dominance |
|  | - | 0 | 0 | AD | -1+0=-1(AD) | NR | Independence |
|  | - | 0 | - | -S | -1+0=(S-)<-1 | NR | Modulation |
|  | - | 0 | -- | -S | -1+0=(S-)<-1 | NR | Modulation |
|  |  |  |  |  |  |  |  |
| Negative neutral | 0 | - | ++ | +S | 0+-1=0<(+S) | NR | Emergence |
|  | 0 | - | + | -A | 0+-1=-1<(A-)≤0 | NR | Dominance |
|  | 0 | - | 0 | AD | 0+-1=-1(AD) | NR | Independence |
|  | 0 | - | - | -S | 0+-1=(S-)<-1 | NR | Modulation |
|  | 0 | - | -- | -S | 0+-1=(S-)<-1 | NR | Modulation |
|  |  |  |  |  |  |  |  |
| Positive neutral | + | 0 | ++ | +S | 1+0=1<(+S) | NR | Modulation |
|  | + | 0 | + | +S | 1+0=1<(+S) | NR | Modulation |
|  | + | 0 | 0 | AD | 1+0=1(AD) | NR | Independence |
|  | + | 0 | - | +A | 1+0=0≤(A+)<1 | NR | Dominance |
|  | + | 0 | -- | -S | 1+0=(-S)<0 | NR | Emergence |
|  |  |  |  |  |  |  |  |
| Positive neutral | 0 | + | ++ | +S | 0+1=1<(+S) | NR | Modulation |
|  | 0 | + | + | +S | 0+1=1<(+S) | NR | Modulation |
|  | 0 | + | 0 | AD | 0+1=1(AD) | NR | Independence |
|  | 0 | + | - | +A | 0+1=0≤(A+)<1 | NR | Dominance |
|  | 0 | + | -- | -S | 0+1=(-S)<0 | NR | Emergence |
|  |  |  |  |  |  |  |  |
| Double neutral | 0 | 0 | ++ | +S | 0+0=0<(+S) | NR | Emergence |
|  | 0 | 0 | + | +S | 0+0=0<(+S) | NR | Emergence |
|  | 0 | 0 | 0 | AD | 0+0=0(AD) | NR | Equivalence |
|  | 0 | 0 | - | -S | 0+0=(-S)<0 | NR | Emergence |
|  | 0 | 0 | -- | -S | 0+0=(-S)<0 | NR | Emergence |
